# Evaluation of data availability on population health indicators at the regional level across the European Union

**DOI:** 10.1186/s12963-019-0188-6

**Published:** 2019-08-07

**Authors:** Claudia Costa, Ângela Freitas, Iwa Stefanik, Thomas Krafft, Eva Pilot, Joana Morrison, Paula Santana

**Affiliations:** 1 Centre of Studies in Geography and Spatial Planning (CEGOT), Department of Geography and Tourism, University of Coimbra, 3004-530 Coimbra, Portugal; 2 Faculty of Health, Medicine and Life Sciences (FHML), Care and Public Health Research Institute (CAPHRI), Department of Health, Ethics and Society, Maastricht University, Maastricht, The Netherlands; 3 Research Department of Epidemiology and Public Health, University College London, London, UK

**Keywords:** Population health, Data availability, Indicators, European regions, NUTS, EURO-HEALTHY, Statistics, Policies

## Abstract

**Background:**

The ability to measure regional health inequalities across Europe and to build adequate population health indices depends significantly on the availability of reliable and comparable data at the regional level. Within the scope of the EU-funded project EURO-HEALTHY, a Population Health Index (PHI) was built. This model aggregates 39 indicators considered relevant by experts and stakeholders to evaluate and monitor population health on the regional level within the European Union (269 regions). The aim of this research was to assess the data availability for those indicators. As a subsequent aim, an adequate protocol to overcome issues arising from missing data will be presented, as well as key messages for both national and European statistical authorities meant to improve data collection on population health.

**Methods:**

The methodology for the study includes three consecutive phases: (i) assessing the data availability for the respective indicators at the regional level for the last year available (ii) applying a protocol for missing data and completing the database and (iii) developing a scoring system ranging from 0 (no data available; worst) to 1 (all data available; best) to evaluate the availability of data by indicator and EU region.

**Results:**

Although the missing data on the set of the PHI indicators was significant, the mean availability score for the EURO-HEALTHY PHI indicators is 0.8 and the regional availability score is 0.7, which reveal the strength of the indicators as well as the data completeness protocol for missing data.

**Conclusions:**

This study provides a comprehensive data availability assessment for population health indicators from multiple areas of concern, at the EU regional level. The results highlight that the data completeness protocol and availability scores are suitable tools to apply on any indicator’s data source mapping. It also raises awareness to the urgent need for sub-national data in several domains and for closing the data gaps between and within countries. This will require policies clearly focused on improving equity between regions and a coordinated effort from the producers of data (the EU28 national statistics offices and EUROSTAT) and the stakeholders who design policies at EU, regional and local level.

**Electronic supplementary material:**

The online version of this article (10.1186/s12963-019-0188-6) contains supplementary material, which is available to authorized users.

## Background

Evidence of a widening health gap between EU countries and regions [1–3] requires the ability to measure and monitor indicators that may reveal inequalities [4–6], in line with a public health perspective where populations from the same region share similar conditions that directly or indirectly affect their health [7].

To better understand why some populations are healthier than others and to take action which will improve health and reduce inequalities, monitoring should go far beyond measuring health outcomes [8]. Within the well-known Dahlgren and Whitehead ‘social model of health’ [9], health inequalities are commonly analysed across several dimensions relating to social, economic and environmental determinants. Many recognise that to reduce inequalities in mortality and morbidity, a shift in health monitoring is required, including the causes and risk factors that continue to cause many communities to lag behind when it comes to the concept of population health in its broadest sense [10–14].

Indicators are well-established monitoring tools, not only for their ability to measure but more specifically because they enable priorities to be set, policies to be formulated and said policies to be evaluated [15–19]. The task of monitoring population health inequalities using indicators from multiple dimensions calls for the availability of spatially disaggregated data at various levels. Having sound data is thus vital to identify gaps and better understand policy impacts, which will enhance informed decision-making [5, 19–26].

The availability of data is regarded as an indispensable standard when measuring health inequalities across countries and regions, as it is frequently identified as the inherent challenge in many EU public health projects, conferences and meetings [13, 23, 25, 27–30]. Sustainable development goals (SDGs) also support the need for data by including an appeal to countries to increase the availability of disaggregated data as part of the goal to strengthen data monitoring and accountability (SDG target 17.18) [6, 8, 19].

The availability of data is a key component of indicator quality assessment [8, 31, 32]. It is defined as the degree of convenience for users to obtain data and related information [32], as it includes the difficulty level that users may experience when accessing data (e.g. whether the data is public or easy to purchase) and its timeliness (e.g. whether data are regularly updated) [32–34]. Reliability is another key component used, and it refers to ‘whether we can trust the data’ [32]. Frequently, data quality is described in terms of its completeness (a reliability element), considering the existence or not of a specific data component (e.g. missing values for a year or region). There are different characteristics and ways of assessing indicator data quality reported in the literature: (i) timeliness and frequency of data updates [35], (ii) data availability at a specific geographical scale [36], (iii) relevance of the data according to the user’s needs and in terms of indicator definition [37] and (iv) multiple data quality components that are used to build a composite index in which indicator quality is assessed under a scoring system [23, 24, 38, 39].

Over the past four decades, the EU has made substantial progress in developing and improving the data quality of population health indicators at the national level [40] with respect to monitoring policies dealing with the environment [41], road safety [42], housing [43], education [44, 45], social protection and inclusion [46], social cohesion [47, 48] and economic development [49]. The EU Public Health monitoring and reporting system is an example of this effort, along with the multi-phase action ‘European Community Health Indicators’ (ECHI, ECHI 2 and ECHIM) [26, 28, 36] and the two-phase project ‘Health Indicators in the European Regions’ (ISARE and I2SARE), which introduced the monitoring at sub-national levels [24, 39, 50–52].

Within the EU, the Nomenclature of Territorial Units for Statistics (NUTS) provides a common standard for data collection and statistical purposes, with the NUTS 2 level designation used by the European Commission for the allocation of Cohesion Funds. In this context, having available and comparable data at NUTS 2 level is fundamental to better understand the challenges and opportunities of each region [53]. However, despite various efforts*,* the lack of regionalised, reliable and comparable data on relevant dimensions to evaluate population health continues to represent a challenge for measuring and monitoring regional health inequalities [13, 24, 39, 50, 51].

The goal of the EU research project ‘Shaping European policies to promote Health Equity’ (EURO-HEALTHY)^1^ was to overcome this lack of health-related data across EU regions. It sought to advance knowledge on policies with the greatest potential to promote health and health equity across EU regions. Underlying this project is the use of multi- and trans-disciplinary approaches and methods to analyse health inequalities. A multidimensional measure—the EURO-HEALTHY Population Health Index (PHI)—was developed to evaluate EU population health across multiple dimensions and at the regional level (269 NUTS 2^2^ from the 28 EU countries) and for the reference year of 2014. The underlying approach of this project, described as a ‘population health approach’, defines population health considering both health outcomes and health determinants, and the policies that influence the optimal balance of determinants [55, 56]. Following this ground-breaking and integrated concept of population health [55–57], the PHI includes multiple indicators of health determinants and health outcomes [58, 59]. It is based on a hierarchical evaluation model structure [60, 61] where the population health of each EU region can be analysed in an aggregated or disaggregated way over a wide range of areas of concern: (i) economic conditions, social protection and security; (ii) education; (iii) demographic change; (iv) lifestyle and health behaviours; (v) physical environment; (vi) built environment; (vii) road safety; (viii) healthcare resources and expenditure; (ix) healthcare performance; and (x) health outcomes [62]. An area of concern reflects broad values of interest to analyse population health and its inequalities, integrating a set of independent evaluation axes (dimensions) which in turn are made operational by means of one or more indicators. The set of indicators in each area of concern was selected via a web-based Delphi process, involving an international and multidisciplinary panel of experts and stakeholders, who stated their views on the extent to which an indicator was relevant for characterising population health [54].

Having a consistent overview of inequalities in health determinants and health outcomes between EU regions requires the completeness of data in all indicators as a main assumption to apply a hierarchical evaluation model structure [63]. The aim of this paper is to assess the data availability of the 39 EURO-HEALTHY PHI indicators considered relevant by experts and stakeholders for evaluating and monitoring population health within the European Union on the regional level. As a subsequent aim, an adequate protocol to overcome the issues of missing data will be presented, as well as key messages to the national and European statistical authorities for improving data collection on population health. Therefore, research reported in this article follows the phase of defining and selecting indicators [54] yet precedes the PHI modelling phase [64] as it is centred on the data collection and data quality assessment of the 39 indicators of the index.

## Methods

### Data collection

The data collection of the indicators to be included in the EURO-HEALTHY PHI was done between November 2015 and July 2016, mainly using major international data sources (EUROSTAT and WHO), considering data for the period 2000–2015 and at the regional level (for all 269 NUTS 2). This geographic scale of analysis was chosen as it is the statistical unit applied by the European Structural and Investment Funds (ESIF) to determine geographic eligibility for funding and to provide essential opportunities to address and invest in interventions that tackle health inequalities across the EU NUTS 2 regions [65]. Data were stored in a PostgreSQL relational database and made available to the public through a web platform: www.eurohealthydata.uc.pt.

### Data completeness

**Fig. 1:**
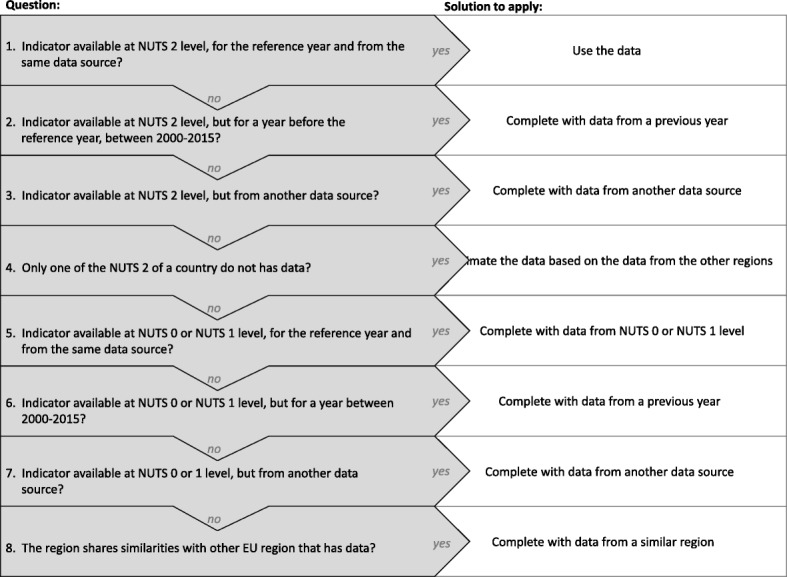
Data completeness protocol. Each rectangle represents the subsequent binary question used to complete the data. Two answers can be considered: yes or no. If the answer to the question is yes, the white square gives the instruction regarding how to complete the data. If the answer is no, the following question must be made

An exploratory analysis was undertaken for each indicator in order to identify whether there were data gaps considering the geographical scale (NUTS 2 level), reference year (2014) and data source (for each indicator, a reference data source was defined). Figure 1 shows the protocol used to check data availability and to overcome potential cases of missing data. A protocol with eight straight binary questions was applied in case of having no data. It is focused on the three pre-established criteria on data availability: (i) at NUTS 2 level, (ii) for the year 2014 and (iii) from the reference data source. For the cases where it was impossible to retrieve data either from another geographical level or from another year or data source, values could be (i) estimated, considering the population distribution and the values of other NUTS 2 regions within the same country; or (ii) assigned, considering values from other region or country sharing similar geographical, political, social and economic characteristics. Additional file 1 presents a more detailed flowchart of the logical decisions taken to complete the data.

### Evaluation criteria and development of a scoring system

A scoring system, ranging from 0 (data not available) to 1 (all data available), was applied to evaluate overall data availability according to two groups of criteria (Table 1).

**Table 1:** Scoring system used to assess the data availability of the EURO-HEALTHY PHI by indicator and Region

Score	Regions/Indicators (%)				
	1. Data available at NUTS 2 level	2. Data available for the reference year	3. Data available from the same data source	4. Estimated data	5. Data from a similar NUTS 2/Country
1	100%	100%	100%	0%	0%
0.8	80–99%	80–99%	80–99%	1–19%	1–19%
0.6	60–79%	60–79%	60–79%	20–39%	20–39%
0.4	40–59%	40–59%	40–59%	40–59%	40–59%
0.2	20–39%	20–39%	20–39%	60–79%	60–79%
0.1	1–19%	1–19%	1–19%	80–99%	80–99%
0	0%	0%	0%	100%	100%
Groups of criteria	Group I			Group II	

Reading example: if an indicator has 85% of data from the reference year and 22% of data estimated, the availability score for criteria 2 will be 0.8 and for criteria 4 it will be 0.6

Group I includes the criteria related to three mandatory data components (geographical scale, year and data source), while group II corresponds to elective data (estimated or assigned values from a similar NUTS 2 /Country). A higher weight (0.7 in 1) was attributed to criteria from group I when compared with group II (0.3), given the higher relevance of overcoming data gaps in mandatory data components.

The score was applied to each indicator and to each NUTS 2 region, resulting in two independent scores: the indicator’s availability score and the regional availability score. The first revealed which indicator presented more data gaps across EU regions, and the second showed which regions and countries have less data available.

The final score was calculated as follows:

1 $$ \mathrm{Final}\ \mathrm{score}=\left(\frac{\mathrm{Criteria}1+\mathrm{Criteria}2+\mathrm{Criteria}3}{3}\ast 0.7\right)+\left(\frac{\mathrm{Criteria}4+\mathrm{Criteria}5}{2}\ast 0.3\right) $$

An indicator’s availability score of 1 means that the indicator is available for all the regions for the same reference year and the same data source. Similarly, a regional availability score of 1 indicates that all 39 indicators are available for that region for the respective reference year and data source.

The score is analysed in six classes, coloured from orange to green, using the following cut-offs: 0.2, 0.4, 0.6, 0.8, 0.9 and 1.0.

## Results

### Data availability of Population Health Index indicators

The EURO-HEALTHY Population Health Index integrates 39 indicators that measure population health inequalities across ten areas of concern and 17 dimensions of Health Determinants and Health Outcomes [62]. Data was collected from official statistics, with the EUROSTAT database comprising 80% of the indicators and the WHO Health for all database (WHO/Europe) a total of 15% (Table 2). A significant proportion (35%) was built with derived data (e.g. PM_2.5_ concentrations; Health personnel; Amenable deaths due to healthcare). When considering the geographical scale, it was found that a significant number of indicators considered relevant to characterise population health are available only at the country level. On average, 74% of the data from indicators produced at NUTS 2 level is available. For indicators produced at the country level, it is 82%.

**Table 2:** Data availability for the EURO-HEALTHY PHI Indicators, according to the geographical scale and reference year

Component	Dimension	Indicator	Source	Geographical scale	Reference year	Data availability (%)	
						NUTS2 (*n* = 269)	Country (*N* = 28)
Health Determinants	Area of concern: Economic conditions, social protection and security						
	Employment	Unemployment rate (%)	EUROSTAT	NUTS 2	2014	99.6	
		Long-term unemployment rate—12 months and more (%)	EUROSTAT	NUTS 2	2014	97.4	
	Income and living conditions	Disposable income of private households per capita (Euro per inhabitant)	EUROSTAT	NUTS 2	2012	99.3	
		People at risk of poverty or social exclusion (%)	EUROSTAT	NUTS 2	2014	21.9	
		Disposable income ratio—S80/S20 (ratio)	EUROSTAT	Country	2014		92.9
	Social protection	Expenditure on care for elderly (% of GDP)	EUROSTAT	Country	2008		96.4
	Security	Crimes recorded by the police per 100.000 inhabitants	EURO-HEALTHY/EUROSTAT	NUTS 2	2010	65.4	
	Area of concern: Education						
	Education	Population aged 25–64 with upper secondary or tertiary education attainment (%)	EUROSTAT	NUTS 2	2014	99.3	
		Early leavers from education and training (%)	EUROSTAT	NUTS 2	2014	93.3	
	Area of concern: Demographic change						
	Ageing	Rate of older people at risk of poverty—aged 65 years or over (%)	EUROSTAT	Country	2013		92.9
		Ageing index (ratio)	EURO-HEALTHY/EUROSTAT	NUTS 2	2014	100.0	
	Area of concern: Lifestyle and health behaviours						
	Lifestyle and health behaviours	Adults who are obese (%)	EUROSTAT	Country	2008		96.4
		Daily smokers—aged 15 and over (%)	OECD	Country	2013		35.7
		Pure alcohol consumption—aged 15 and over (litres per capita)	HFA-DB	Country	2013		28.6
		Live births by mothers under age of 20 (%)	EURO-HEALTHY/EUROSTAT	NUTS 2	2013	85.9	
	Area of concern: Physical environment						
	Pollution	Annual mean of the daily PM2.5 concentrations (μg/m^3^)	EURO-HEALTHY/EEA	NUTS 2	2011	98.9	
		Annual mean of the daily PM10 concentrations (μg/m^3^)	EURO-HEALTHY/EEA	NUTS 2	2011	98.9	
		Greenhouse Gas (GHG), total tonnes of CO_2_ eq. emissions per annum per capita	EUROSTAT	Country	2012		100.0
	Area of concern: Built environment						
	Housing conditions	Average number of rooms per person	EUROSTAT	NUTS 2	2014	35.3	
		Households without indoor flushing toilet (%)	EURO-HEALTHY/EUROSTAT	NUTS 2	2011	97.4	
		Households without central heating (%)	EURO-HEALTHY/EUROSTAT	NUTS 2	2011	96.7	
	Water and sanitation	Population connected to wastewater treatment plants	EUROSTAT	Country	2014		25.0
		Population connected to public water supply	EUROSTAT	NUTS 2	2013	14.5	
	Waste management	Recycling rate of municipal waste (%)	EUROSTAT	Country	2013		100.0
	Area of concern: Road safety						
	Road safety	Victims in road accidents—injured and killed, per 100,000 inhabitants	EURO-HEALTHY/EUROSTAT	NUTS 2	2013	91.5	
		Fatality rate due to road traffic accidents, per 1000 victims	EURO-HEALTHY/EUROSTAT	NUTS 2	2013	91.5	
	Area of concern: Healthcare resources and expenditure						
	Healthcare resources	Medical doctors, per 100,000 inhabitants	EUROSTAT	NUTS 2	2013	61.4	
		Health personnel (nurses and midwives, dentists, pharmacists and physiotherapists), per 100,000 inhabitants	EURO-HEALTHY/EUROSTAT	NUTS 2	2013	44.6	
	Healthcare expenditure	Total health expenditure (THE), PPP$ per capita, WHO estimates	HFA-DB	Country	2013		100.0
		Private households’ out-of-pocket on health as percentage of total health expenditure (THE)	HFA-DB	Country	2013		100.0
		Public expenditure on health, PPP$ per capita, WHO estimates	HFA-DB	Country	2013		100.0
	Area of concern: Healthcare performance						
	Healthcare performance	Hospital discharges due to diabetes, hypertension and asthma, per 100,000 inhabitants	EURO-HEALTHY/EUROSTAT	NUTS 2	2013	32.7	
		Amenable deaths due to health care—standardised death rate, per 100,000 inhabitants	EURO-HEALTHY/EUROSTAT	NUTS 2	2011–13	38.3	
Health Outcomes	Area of concern: Health outcomes						
	Mortality	Life expectancy at birth (years)	EUROSTAT	NUTS 2	2013	85.9	
		Infant mortality, per 1000 live births	EUROSTAT	NUTS 2	2011–13	99.6	
		Preventable deaths - standardised death rate, per 100,000 inhabitants	EURO-HEALTHY/EUROSTAT	NUTS 2	2011–13	24.9	
	Morbidity	Self-perceived health less than good (%)	EUROSTAT	Country	2013		100.0
		Age-standardised disability-adjusted life year (DALY) rates	HFA-DB	Country	2012		100.0
		Low birth-weight (%)	EURO-HEALHTY/ HFA-DB/ EUROSTAT	Country	2013		64.3

For indicators collected directly from official data sources, the name of the statistics producer appears in the ‘source’ column. For the indicators derived from data collected from official data sources, EURO-HEALTHY and the name of the statistics producer appear in the ‘source’ column

The table presents two different things: (1) the PHI model structure with the PHI’s components, areas of concern, dimensions and indicators; and (2) General information about the indicators as to where they are available

### Data completeness of the Population Health indicators

**Fig. 2:**
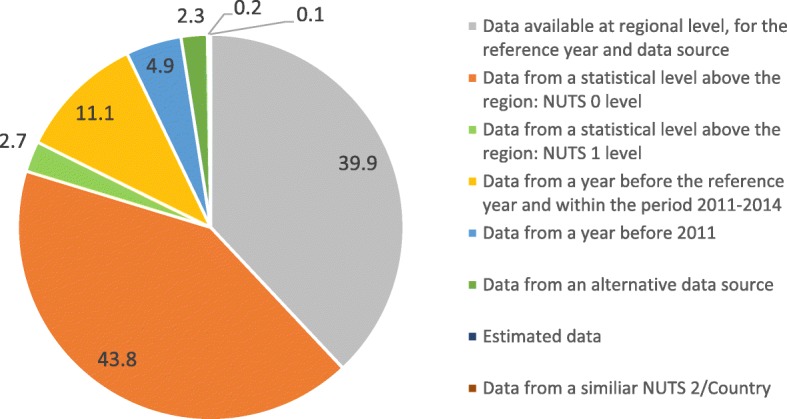
Source of the data required to complete the Population Health Indicators. The graph represents how the missing data was completed and the share of each solution used to complete the data

More than half of the data required to build the Population Health Index was unavailable with respect to the criteria of having NUTS 2 level data for the reference year and from the reference data source. Whenever gaps in the available statistical data were found, other data were used to fill in the gaps (Fig. 2). Most of this data came from a statistical level above the region or from a previous year.

### Analysis of the indicator’s and regions’ availability scores

**Fig. 3:**
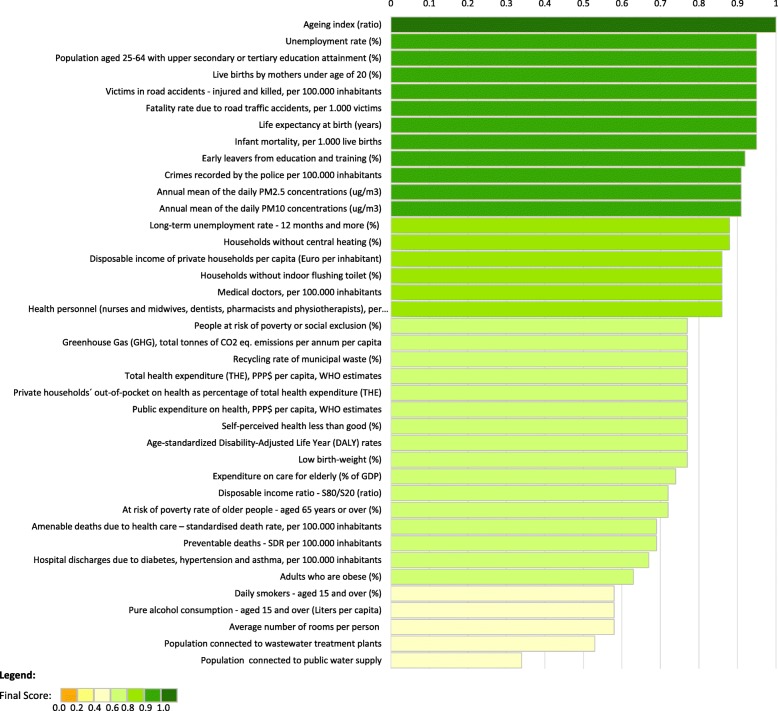
Ranking of the indicators, by availability score. The graph represents the final availability score of each indicator. The indicators are organised on a ranking. The colour of the bars represents the score categories

The application of the indicator’s availability score to the set of indicators resulted in an overall mean score of 0.79, ranging from 0.46 to 1.00 (Fig. 3). Additional file 2 presents the scores by area of concern, dimension and indicator by criteria. The analysis showed that the higher mean scores (above 0.90) belong to the dimensions of Employment, Education, and Road safety. The lowest mean scores were found in the dimensions of Water and sanitation (0.50), Lifestyle and health behaviours (0.69) and Healthcare performance (0.68). The lowest mean scores were, for the most part, associated with lack of data at the NUTS 2 level (mean score = 0.46) and reference year (mean score = 0.75).

**Fig. 4:**
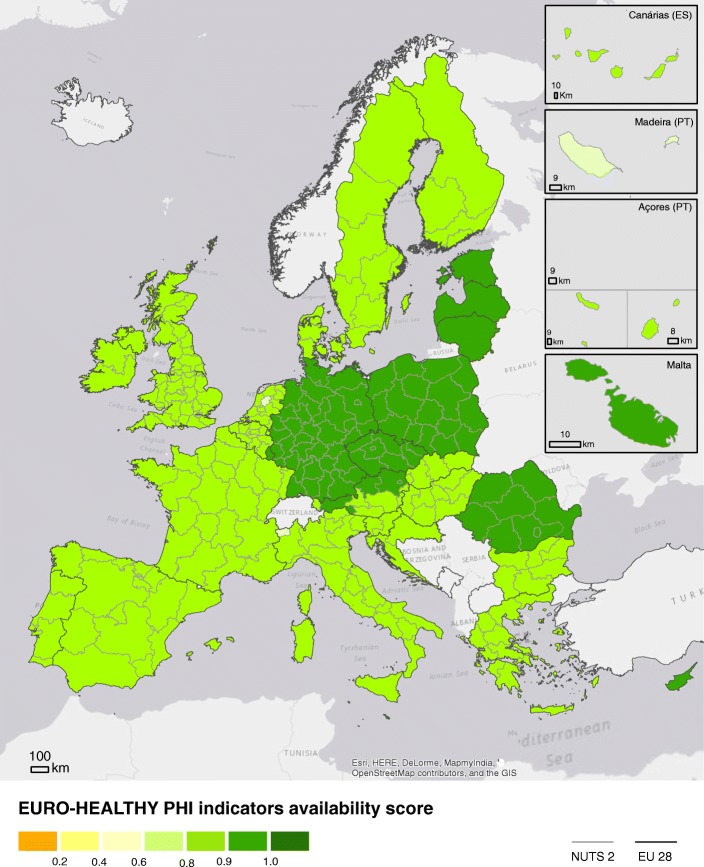
Map of overall regional availability score in the EU28. The colours represent the score achieved by each region within EU28 NUTS 2 level. Green colours represent higher availability. Orange colours represent lower availability

Figure 4 displays the geographical distribution of the regional availability score applied to all 269 NUTS 2 regions. The overall mean score was 0.71. Although no region reached the optimal score of 1, the map shows that almost all the NUTS 2 (73.2%) registered high mean scores (above 0.75), with Lithuania and Luxembourg (single-region countries) presenting the highest scores (0.86) followed by regions in Austria, the Czechia and Sweden. At the opposite end of the scale, regions located in Croatia, Ireland, France, Finland, and certain regions of the UK performed worse due to the lack of data in important data assessment criteria.

Similar to the indicator’s availability score, regions performing worse are those presenting lack of data at NUTS 2 level (mean score = 0.42) and for the reference year (mean score = 0.74). The analysis by area of concern, available in Additional file 3, revealed that a large number of regions are lacking data in one or more criteria of group 1, namely within Lifestyle and health behaviours, Healthcare performance and Built environment, which yielded the lowest mean scores (0.63, 0.69 and 0.69, respectively). A high level of internal variability was found in the areas of concern of Healthcare performance and Health outcomes, where within the same country there are regions presenting different mean availability scores.

## Discussion

To our knowledge, this study is the first of its kind to assess data availability of population health indicators for all the 269 EU regions and to identify the adequate protocol to overcome issues with missing data without compromising the quality of the Population Health Index.

Key take-away messages that summarise the main results and their implications for further research and aim to improve data collection at sub-national level across EU will drive the discussion: (1) Data completeness protocol and availability scores are suitable tools to apply on any indicator’s data source mapping; (2) Overcoming missing data issues should be a priority; and (3) Data collection is driven by policy.

### Data completeness protocol and availability scores are suitable tools to apply on any indicator’s data source mapping

Indicators are well-established monitoring tools. Thus, applying a data source mapping and analysing their availability is an essential initial step for monitoring population health inequalities [8]. Due to weaknesses identified in this step, indicators are often removed from the analysis [66] or the purpose of the study is compromised [23].

The data collection of the set of 39 EURO-HEALTHY PHI indicators, from 17 different dimensions, encountered challenges, particularly those related to assuring that the indicators were available for all the EU regions and for the same year of analysis. The application of a data completeness strategy allowed for filling in the existent data gaps, resulting in a relatively high score, both at the indicator and regional levels (0.8 and 0.7, respectively, in a range from 0 to 1). This protocol, based on single strategies previously defined [19], allowed for the construction of the EURO-HEALTHY Population Health Index. Otherwise, it would not be possible to cover all EU regions and some indicators would be excluded.

The EURO-HEALTHY PHI is seen as a step forward, one which raises awareness of the lack of relevant data to monitor population health and represents the effort to provide an integrated assessment of health (considering indicators of Health Outcomes and Health Determinants) and a geographically meaningful tool allowing for the analysis and comparison of health across all regions of the 28 EU countries in a specific year. The final purpose underlying the development of this tool is to use its capabilities to inform regional policies by providing evidence on relevant dimensions where policy action has high potential for reducing inequalities in health between regions [62]. As it is widely understood in the public health community, no data, no knowledge, no action [67]. When developing an index, obstacles and constraints arise when dealing with the availability of data on indicators considered relevant to inform policies.

Through the PHI model, the EURO-HEALTHY project already defined the framework for monitoring population health in Europe. So, for the future, it is important to continue evaluating data availability and discussing the data collection process at EU level.

### Mind the health gap: why overcoming missing data should be a priority

The first assumption of a good monitoring system of health inequalities across Europe is to have good quality data, which is available and comparable between different countries and regions [8]. According to the literature, having sound data is vital to identify gaps and better understand policy impacts, which enhances informed decision-making [5, 19–26]. This is particularly challenging when there are countries with different political attitudes towards health inequalities within the EU, from measuring health inequalities to recognising disparities and their consequences on health [68].

The analysis made by dimension revealed significant differences in the indicator’s availability scores, ranging from 0.95 on Road safety (almost all data were available on its indicators) to 0.50 on Water and sanitation (with huge data gaps at the geographical level of NUTS 2).

Availability at NUTS 2 level was the criteria achieving the lowest score, particularly for the indicators of Built environment, Lifestyle and health behaviours and Healthcare performance. Four reasons were identified: (1) indicators’ availability only at the country level, (2) isolated regions with small number of people and specific monitoring systems, (3) lack of adherence between the local and regional monitoring services and the administrative levels supported by EUROSTAT and (4) absence of cohesion between countries on monitoring topics.

A significant number of indicators considered relevant to monitor population health in the EU at regional level are only available at country level. Most of them belong to the European Core Health Indicators (ECHI), which includes the indicators considered as relevant for monitoring national progress in relation to Europe 2020 objectives [69]. Yet, at the sub-national level, they are not routinely collected or even readily available [24, 28]. This is due to a centralised health sector (e.g. health expenditure) or to the data collection process, based on self-reports, developed for a sample representative of the country (e.g. morbidity indicators) and often not comparable for benchmarking (e.g. lifestyle and health behaviour) [24, 36, 39]. For instance, only recently have EU member states been obliged to collect data on health status, along with the provision of healthcare, health determinants and socio-economic conditions of their populations, in the context of the European Health Interview Survey (EHIS) [46, 70]. Prior to this, most countries collected their own data on lifestyles and health behaviours at the regional level through National Health Surveys. Still, countries decide when to collect this data and which indicators are to be collected and disseminated. Therefore, the data present a large variation in terms of the reporting year, and a lack of harmonisation and comparability between countries (e.g. different definition of the survey sampling), so it is not possible to use these National Health Surveys.

Our study also found NUTS 2 regions without data on dimensions with high availability scores like Education and Employment, requiring them to be completed with estimated data. These often corresponded to isolated areas with low number of inhabitants and specific monitoring systems adapted to the local circumstances (e.g. Finland).

The lack of adherence to the EU statistical levels is visible in indicators related with healthcare resources (e.g. Medical doctors, Health personnel) and outcomes (e.g. Hospital discharges) which had to be completed with values at country level. In the past, the ECHI project already identified the performance of healthcare systems as one of the domains requiring extra investment on developing comparable statistics at the regional level [28], and the ISARE project even suggested the use of different geographical levels in order to analyse health data in Europe [24]. Differences between the NUTS classification, used by the EUROSTAT, and the national health regions explain this [24, 39]. According to Wilkinson and colleagues [24], there is a lack of adherence of the health regions to the NUTS level in the ‘old’ countries of the European Union, motivated by a decentralised system where policy-making is at the local level (e.g. Germany and the UK) [71].

The absence of agreement between countries also compromises some indicators associated with built environment and access to water and sanitation. Although EUROSTAT launched a new platform to give access to census data, the European Census Hub tool, few topics are covered due to differences between countries. For instance, the water and sanitation indicators (scores of 0.53 and 0.46) are not yet available via the European Census Hub database due to the lack of comparability across EU countries. Even where data for NUTS 2 level is theoretically available, which is the case of Population connected to public water supply, data at this level appear only to be available for the regions of Eastern European countries, possibly because they were the last to become part of the EU and required substantial investment in improving the levels on access to basic infrastructures. Most of the ‘old’ EU countries already have very high shares of the population connected to water and wastewater treatment plants, which in turn, could potentially explain the cases of missing data for the recent years.

In fact, the temporal scale of the data was the second most applied criteria in order to complete missing data. Countries like Belgium, Denmark and Sweden present data from healthcare resources from a year before the reference year of this study. This is linked to EUROSTAT’s data delivery: EU countries may provide their annual data at any time between 18 to 24 months after the reporting period, so EUROSTAT quite often releases its data for a new year, whereas most countries haven not reported it yet.

Finally, the regional availability score also reveals that none of the EU regions presented data for all 39 indicators according to the criteria, that is, for the regional level (NUT 2 level), for the reference year and for the same data source. This score ranges from 0.91 on Road safety (almost all regions available according to the criteria) to 0.63 on Lifestyles and health behaviours (with most indicators at country level). Surprisingly, the lowest scores identified in our study were found in countries from Central and Western Europe (e.g. France). However, a previous study considering data availability at country level concluded that data availability did not differ between the EU-15 and the EU-27 [36].

### Data collection is driven by policy

Within the European Union, data collection is driven by a policy derived from an international or EC initiative with focus on performance measurement and results-based policy making, stating the framework of indicators to be collected and for which scale [19].

Road safety, Education, Employment and Pollution dimensions reveal indicators with good data availability (above 0.86), which is linked to EU policy development over the years requiring monitoring data at sub-national level [42, 45, 50, 72] to define policies able, for example, to decrease road accidents, reduce the number of early school leavers and achieve high educational levels [44, 73, 74]. The same is revealed by the mortality dimension. EUROSTAT has a long tradition of providing access to mortality indicators [24] due to a number of important EU policies deploying mortality indicators for planning actions, and for monitoring and evaluating programmes, notably in the health, social and economic fields [28, 75].

Still, the argument of EU policy development driving data collection and comparability at sub-national levels do not seem to apply to all policies. Although it is recognisable by the European Commission (EC) that the regional and local level have a crucial role to play in decreasing Greenhouse gas emissions [76], promoting social inclusion [46, 77] and tackling obesity [70], the data collection occurs at the country level. Regarding the monitoring of Greenhouse gas emissions, a region’s lack of capacity to efficiently monitor and observe greenhouse gases is an issue [78]. As for measuring material deprivation and obesity, the EC focuses on analysing demography and social issues at the level of the individual rather than by place of residence, so the data collection frequently relies on survey samples representative only of the country, which limits the capacity to evaluate poverty and obesity issues at sub-national levels [5, 70, 79].

Other policies focused on traffic noise, contaminated sites and exposure to flooding, with impact on population health, do not explicitly mandate the level for data collection [80–82], meaning that such environmental hazards are poorly documented and constitute a significant limitation when informing regional policies.

### Strengths and limitations

Despite the many pitfalls associated with having 39 indicators available for all NUTS 2 regions and for the reference year, this study advances knowledge on the potential of producing a multidimensional database of comparable population health indicators at the EU regional level. The application of a structured and transparent methodology allowed for missing data to be completed, thus adding validity to the database used to build the population health index.

However, six limitations can be identified in this study.

First, the indicators are updated on a regular basis, so the results presented may be seen as outdated rather quickly, meaning that the process has to be frequently updated.

Second, the results of this paper should be interpreted with caution; the regions differ considerably in population size. The NUTS regulation allows for a wide range, between the minimum (800,000 inhabitants) and maximum (3 million) threshold for NUTS 2 regions. This range is simply intended for guidance; there are some NUTS 2 regions with a population less than 30,000 inhabitants (Aland in Finland) and others with over 12 million inhabitants (Île de France in France).

Third, the number of regions in each country contributed substantially to the results, which may represent an important limitation of this study. This happened in cases of data absence for countries with many regions and in single-region countries. Thus, the lack of data at the regional level for the UK (40 NUTS 2) corresponded to almost 15% of missing data at EU level.

Fourth, the weight assigned to each group of criteria has a significant impact on the final score, so different weights would lead to distinct results. Still, the relevance of each criteria is different for the robustness of the PHI, so the final score had to reflect this.

Finally, although we argue that all the indicators included in the PHI should be collected at the regional level and all the data gaps should be tackled, we do not make any reference to the high costs that collecting all this data would entail.

### Further research and recommendations

This article is an attempt to build a bridge between the scientific community and policy-makers. The identification of data gaps at the regional level (NUTS 2) in several areas of concern and dimensions of population health has the potential to inform priorities for data collection and harmonisation. In addition, the findings from the study can (i) advance future research about compiling data for measuring population health under a holistic and multidimensional approach, including health outcomes and health determinants and (ii) highlight important recommendations for both National and European statistical authorities. In addition, they might raise the awareness required to apply the PHI to the entire region of Europe, which would be of relevance in all sub-regions shared across borders within and outside the EU28, where health is co-determined by factors relevant across the borders.

The identification of major data gaps within indicators considered relevant to evaluate population health (included in the PHI) is a call for attention to any future (re)definition of the European statistical system which considers the indicators where data collection is required at the regional level (e.g. built environment). The evaluation of the effectiveness of regional policies in shaping important health determinants demands information and evidence at the sub-national level.

Awareness of the relevance of this data at the regional level can help encourage researchers and other stakeholders to advocate for data collection at several geographical levels. The data availability score developed in this study may have the potential to become a point of departure for decision-makers to assess the quality of the data being used in the monitoring of important indicators which contribute to the improvement of population health.

Finally, a data availability score of 1 would be the goal for all indicators and regions. This would require better coordination on the part of the statistics authorities of each Member State and EUROSTAT to develop high-quality, harmonised and comparable statistics for different geographical levels.

## Conclusions

The challenges encountered in this study underscore the urgent need to close ‘data gaps’ as a condition for closing the ‘health gaps’ within relevant population health indicators between and within EU countries. This is particularly true for health determinants, which are fundamental to inform policy and monitor its effectiveness. This need is mentioned in several international documents and reports, namely the *Health 2020 framework*: *The European policy for health and well-being*, the *European Health Report 2015* [79], the discussion paper on *Closing the gap*: *policy into practice on Social Determinants of Health* [13] and the *Transforming our World*: *The 2030 Agenda for Sustainable Development* [6, 8]. These documents highlight the relevance of the availability of indicators measuring well-being and inequities in population health associated with social determinants, especially at the sub-national level. Public health knowledge on the importance of risk factors and health determinants may be a difficult task in data collection, providing the same attention that is given to monitoring mortality. As a matter of fact, the study reveals that there is still room for improvement.

Notwithstanding, a clear prior statement on tackling regional inequalities within each policy is essential, as the data collection is linked to the policy-making process followed at EU level. Data at the sub-national level is essential for implementing policies that address inequities, but also for better decision-making and accountability at the local level. To ensure that this data will serve as the catalyst for action, it is important to increase awareness that sub-national data promotes better understanding of the baseline levels, information to design effective policies and an explanation of the potential impact of policies. Therefore, it is crucial that progress be made on the link between social determinants, policies and health inequities.

## Additional files

Additional file 1: Data completeness flowchart. Flowchart considered to complete the missing data on the Population Health Indicators. The first step corresponds to the identification of the geographical level the indicator is. If it is available at regional level, the option A must be considered. If it is available at country level, the option B must be applied. (PDF 671 kb)

Additional file 2: Indicator availability score of the EURO-HEALTHY PHI indicators. Table with the availability scores from each indicator and dimension by criteria. (PDF 487 kb)

Additional file 3: Map of regional availability score in the EU, by area of concern. Figure with the regional availability score of each area of concern. (PDF 1870 kb)

## Abbreviations

EC: European Commission
ECHI: Project ‘European Community Health Indicators’
ECHIM: Project ‘European Community Health Indicators Monitoring’
EEA: European Environment Agency
EU: European Union
EURO-HEALTHY: Project ‘Shaping EUROpean policies to promote HEALTH equitY’
ISARE: Project ‘Health Indicators in the European Regions’
MEHM: Minimum European Health Module
NUTS: Nomenclature of Territorial Units for Statistics
OECD: Organisation for Economic Co-operation and Development
OMC: Open Method of Coordination
PHI: Population Health Index

## Footnotes

1
http://www.euro-healthy.eu/

2 The outermost regions and autonomous cities were not included in EURO-HEALTHY project since many of the indicators are not available for these NUTS 2. This concrete anal is beyond the scope of this study. For more information about the process, see the work by Freitas et al. [54].
