# Supplementary material for: Evaluation of data availability on population health indicators at the regional level across the European Union
Source: Popul Health Metr. 2019 Aug 7;17:11. doi: 10.1186/s12963-019-0188-6 (PMC6686464; doi:10.1186/s12963-019-0188-6)
Supplement: Supplementary file 1 — Data completeness flowchart. Flowchart considered to complete the missing data on the Population Health Indicators. The first step corresponds to the identification of the geographical level the indicator is. If it is available at regional level, the option A must be considered. If it is available at country level, the option B must be applied. (PDF 671 kb) [file 12963_2019_188_MOESM1_ESM.pdf]

Additional file 1: Data completeness flowchart

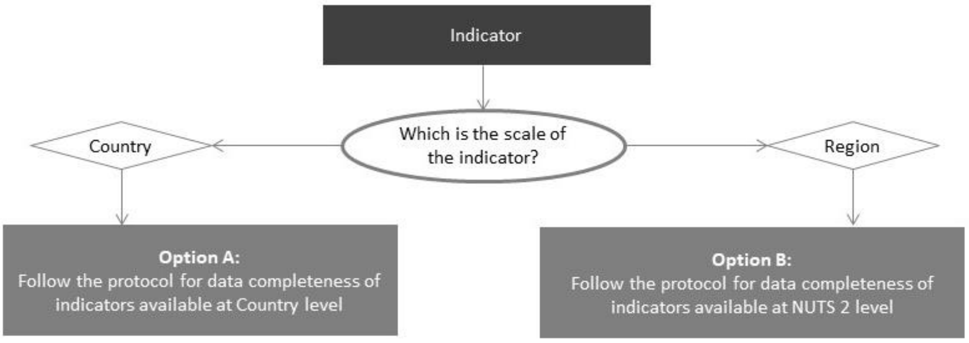

Option A: Protocol for data completeness of indicators available at Country level:

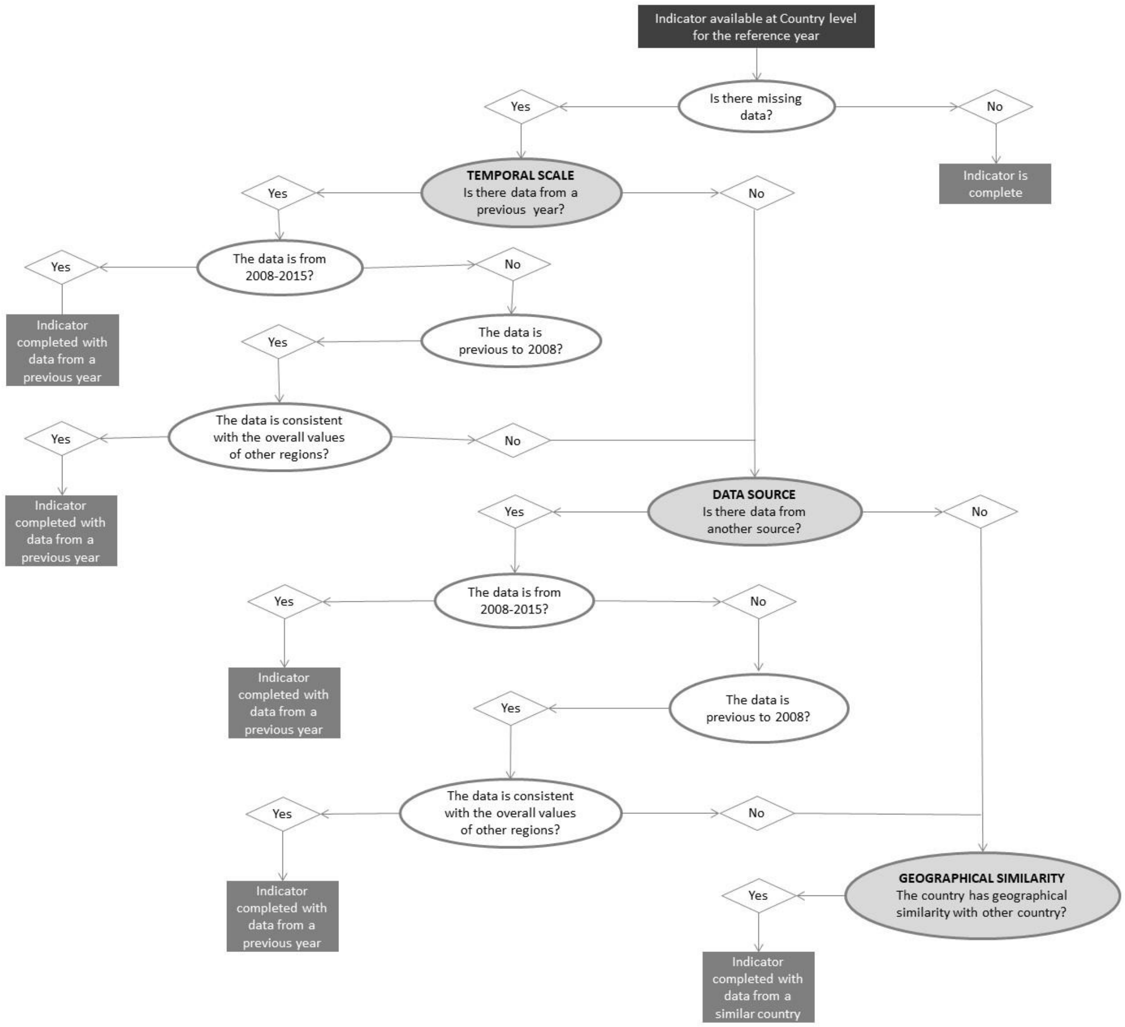

Option B: Protocol for data completeness of indicators available at Regional level:

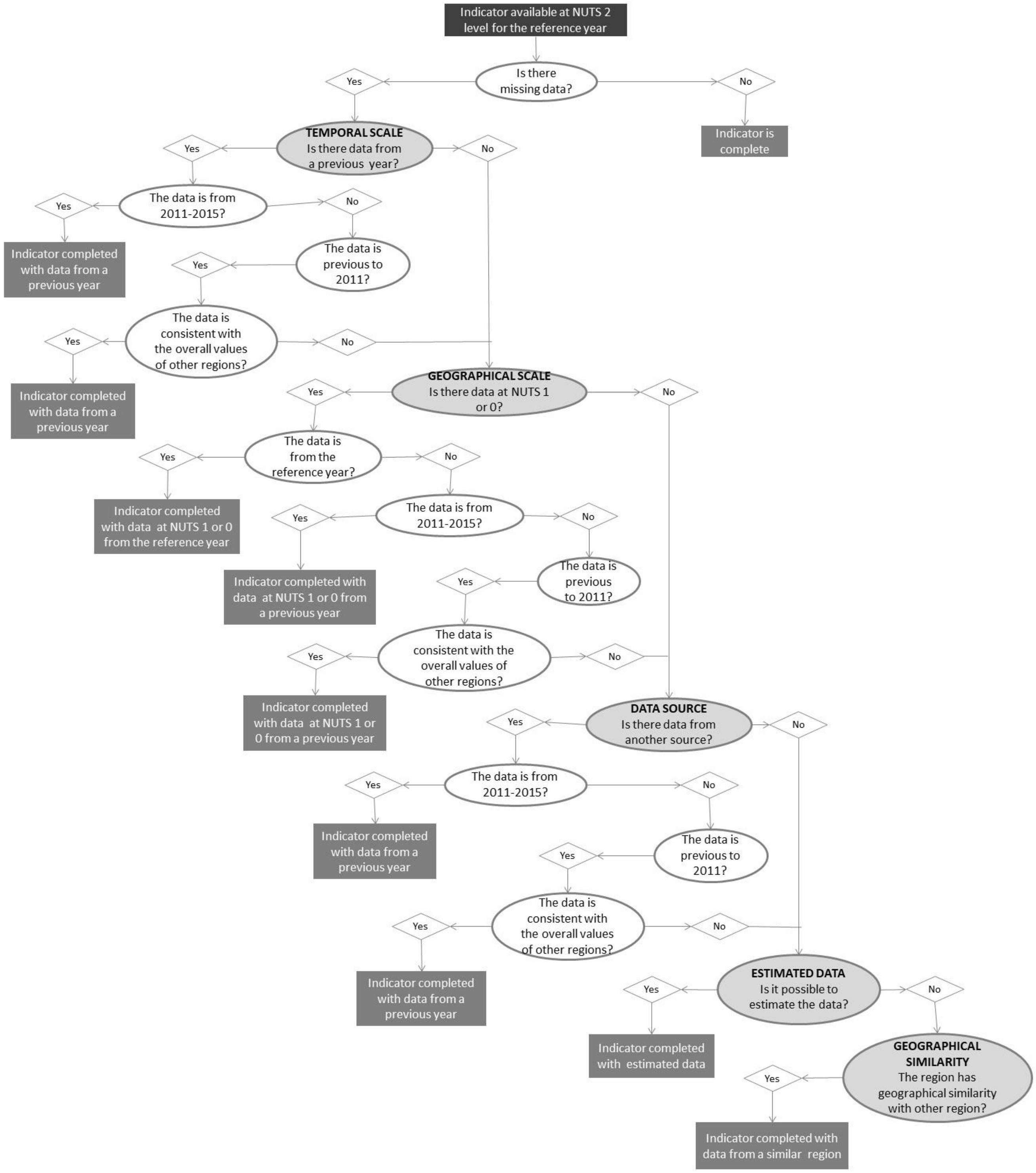

Legend: The grey circles represent the criteria used to complete the data. The white circles represent the subsequent question used to complete the data. The white triangle represents the answer to the previous question (yes or no). The grey squares represent the decision made to complete the data.
