# Supplementary material for: Evaluation of data availability on population health indicators at the regional level across the European Union
Source: Popul Health Metr. 2019 Aug 7;17:11. doi: 10.1186/s12963-019-0188-6 (PMC6686464; doi:10.1186/s12963-019-0188-6)
Supplement: Supplementary file 2 — Indicator availability score of the EURO-HEALTHY PHI indicators. Table with the availability scores from each indicator and dimension by criteria. (PDF 487 kb) [file 12963_2019_188_MOESM2_ESM.pdf]

Additional File 2: Indicator availability score of the EURO-HEALTHY PHI indicators.

Component	Area of Concern	Indicator	Data availability score: Group I: 1. NUTS 2 level	Group I: 2. Reference year	Group I: 3. Data source	Group II: 4. Estimated	Group II: 5. Similar	Final Score
HEALTH DETERMINTNATS COMPONENT	ECONOMIC CONDITIONSSOCIAL PROTECTION AND SECURITY	**Dimension: Employment**	**0.8**	**0.9**	**1.0**	**0.9**	**1.0**	**0.92**
		Unemployment rate (%)	0.8	1.0	1.0	1.0	1.0	0.95
		Long-term unemployment rate - 12 months and more (%)	0.8	0.8	1.0	0.8	1.0	0.88
		**Dimension: Income and living conditions**	**0.3**	**0.8**	**0.9**	**1.0**	**1.0**	**0.78**
		Disposable income of private households per capita (Euro per inhabitant)	0.8	0.8	0.8	1.0	1.0	0.86
		People at risk of poverty or social exclusion (%)	0.2	0.8	1.0	1.0	1.0	0.77
		Disposable income ratio - S80/S20 (ratio)	0.0	0.8	1.0	1.0	1.0	0.72
		**Dimension: Social protection**	**0.0**	**1.0**	**1.0**	**1.0**	**0.8**	**0.74**
		Expenditure on care for elderly (% of GDP)	0.0	1.0	1.0	1.0	0.8	0.74
		**Dimension: Security**	**0.6**	**1.0**	**1.0**	**1.0**	**1.0**	**0.91**
		Crimes recorded by the police per 100,000 inhabitants	0.6	1.0	1.0	1.0	1.0	0.91
	EDUCATION	**Dimension: Education**	**1.0**	**0.8**	**1.0**	**0.9**	**1.0**	**0.94**
		Population aged 25-64 with upper secondary or tertiary education attainment (%)	1.0	0.8	1.0	1.0	1.0	0.95
		Early leavers from education and training (%)	1.0	0.8	1.0	0.8	1.0	0.92
	DEMOGRAPHIC CHANGE	**Dimension: Ageing**	**0.5**	**0.9**	**1.0**	**1.0**	**1.0**	**0.86**
		At risk of poverty rate of older people - aged 65 years or over (%)	0.0	0.8	1.0	1.0	1.0	0.72
		Ageing index (ratio)	1.0	1.0	1.0	1.0	1.0	1.00
	LIFESTYLE AND HEALTH BEHAVIOURS	**Dimension: Lifestyle and Health Behaviours**	**0.3**	**0.5**	**0.9**	**1.0**	**1.0**	**0.69**
		Adults who are obese (%)	0.0	0.6	0.8	1.0	1.0	0.63
		Daily smokers - aged 15 and over (%)	0.0	0.4	0.8	1.0	1.0	0.58
		Pure alcohol consumption - aged 15 and over (Liters per capita)	0.0	0.2	1.0	1.0	1.0	0.58
		Live births by mothers under age of 20 (%)	1.0	0.8	1.0	1.0	1.0	0.95
	PHYSICAL ENVIRONMENT	**Dimension: Pollution**	**0.7**	**0.9**	**0.9**	**1.0**	**1.0**	**0.86**
		Annual mean of the daily $PM_{2.5}$ concentrations (ug/m^3)	1.0	0.8	0.8	1.0	1.0	0.91
		Annual mean of the daily PM_{10} concentrations (ug/m^3)	1.0	0.8	0.8	1.0	1.0	0.91
		Greenhouse Gas (GHG), total tonnes of CO2 eq. emissions per annum per capita	0.0	1.0	1.0	1.0	1.0	0.77
	ENVI RON	**Dimension: Housing conditions**	**0.6**	**0.6**	**0.9**	**1.0**	**0.9**	**0.77**
		Average number of rooms per person	0.2	0.2	0.8	1.0	1.0	0.58

Area of Concern	Indicator	Data availability score					Final Score
		Group I			Group II		
		1. NUTS 2 level	2. Reference year	3. Data source	4. Estimated	5. Similar	
	Households without indoor flushing toilet (%)	0.8	0.8	0.8	1.0	1.0	0.86
	Households without central heating (%)	0.8	0.8	1.0	1.0	0.8	0.88
	**Dimension: Water and sanitation**	**0.1**	**0.2**	**0.6**	**1.0**	**1.0**	**0.50**
	Population connected to wastewater treatment plants (%)	0.0	0.2	0.8	1.0	1.0	0.53
	Population connected to public water supply (%)	0.2	0.2	0.4	1.0	1.0	0.34
	**Dimension: Waste management**	**0.0**	**1.0**	**1.0**	**1.0**	**1.0**	**0.77**
	Recycling rate of municipal waste (%)	0.0	1.0	1.0	1.0	1.0	0.77
ROAD SAFETY	Dimension: **ROAD SAFETY**	**1.0**	**0.8**	**1.0**	**1.0**	**1.0**	**0.95**
	Victims in road accidents - injured and killed per 100,000 inhabitants	1.0	0.8	1.0	1.0	1.0	0.95
	Fatality rate due to road traffic accidents per 1,000 victims	1.0	0.8	1.0	1.0	1.0	0.95
HEALTHCARE RESOURCES AND EXPENDITURE	**Dimension: Healthcare resources**	**0.6**	**0.8**	**1.0**	**1.0**	**1.0**	**0.86**
	Medical doctors per 100,000 inhabitants	0.6	0.8	1.0	1.0	1.0	0.86
	Health personnel (nurses and midwives. dentists. pharmacists and physiotherapists) per 100,000 inhabitants	0.6	0.8	1.0	1.0	1.0	0.86
	**Dimension: Healthcare expenditure**	**0.0**	**1.0**	**1.0**	**1.0**	**1.0**	**0.77**
	Total health expenditure (THE). PPP$ per capita, WHO estimates	0.0	1.0	1.0	1.0	1.0	0.77
	Private households´ out-of-pocket on health as percentage of total health expenditure (THE)	0.0	1.0	1.0	1.0	1.0	0.77
	Public expenditure on health. PPP$ per capita, WHO estimates	0.0	1.0	1.0	1.0	1.0	0.77
HEALTHCARE PERFORMANCE	**Dimension: Healthcare performance**	**0.6**	**0.3**	**0.8**	**0.9**	**1.0**	**0.68**
	Hospital discharges due to diabetes. hypertension and asthma per 100,000 inhabitants	0.4	0.4	0.8	1.0	1.0	0.67
	Amenable deaths due to health care – standardised death rate per 100,000 inhabitants	0.8	0.2	0.8	0.8	1.0	0.69
HEALTH OUTCOMES COMPONENT – HEALTH OUTCOMES	**Dimension: Mortality**	**0.8**	**0.7**	**0.9**	**0.9**	**1.0**	**0.87**
	Life expectancy at birth (years)	0.8	1.0	1.0	1.0	1.0	0.95
	Infant mortality per 1,000 live births	0.8	1.0	1.0	1.0	1.0	0.95
	Preventable deaths - SDR per 100.000 inhabitants	0.8	0.2	0.8	0.8	1.0	0.69
	**Dimension: Morbidity**	**0.0**	**1.0**	**1.0**	**1.0**	**1.0**	**0.77**
	Self-perceived health less than good (%)	0.0	1.0	1.0	1.0	1.0	0.77
	Age-standardized Disability-Adjusted Life Year (DALY) rates	0.0	1.0	1.0	1.0	1.0	0.77
	Low birth-weight (%)	0.0	1.0	1.0	1.0	1.0	0.77
