# Supplementary material for: Evaluation of data availability on population health indicators at the regional level across the European Union
Source: Popul Health Metr. 2019 Aug 7;17:11. doi: 10.1186/s12963-019-0188-6 (PMC6686464; doi:10.1186/s12963-019-0188-6)

Additional file 3: Map of regional availability score in the EU, by area of concern.

Regional availability score by area of concern: Economic conditions, social protection and security

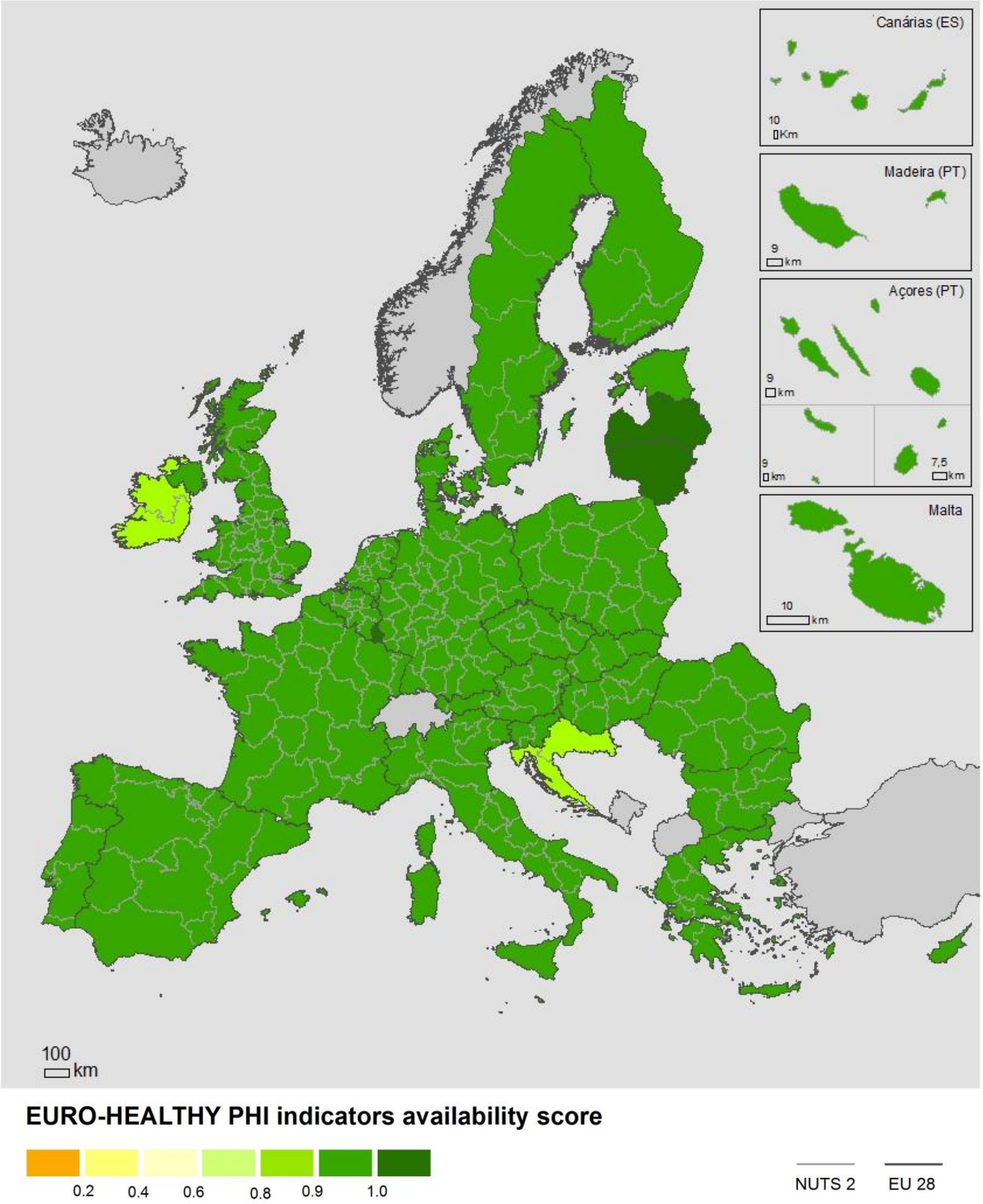

Regional availability score by area of concern: Education

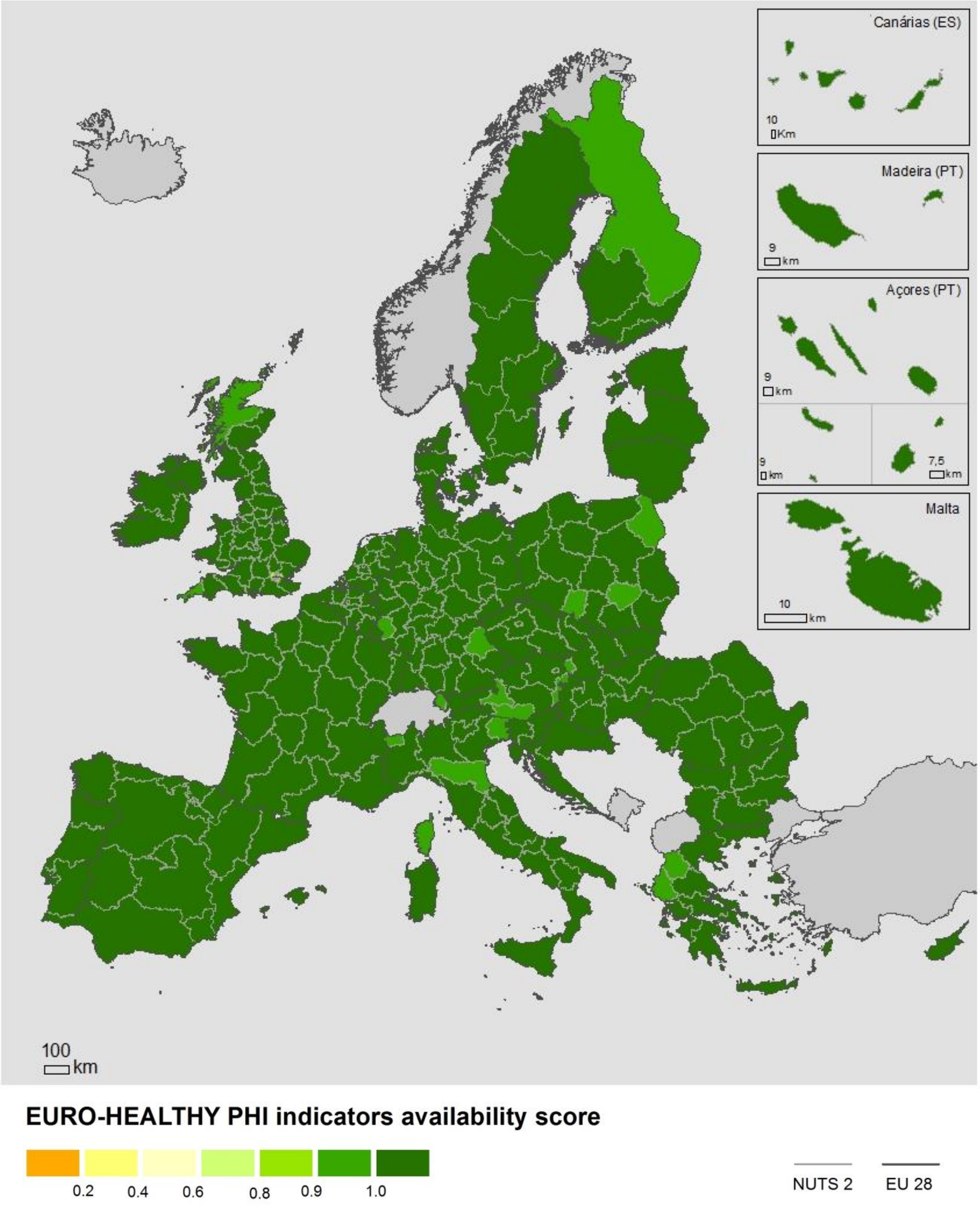

Regional availability score by area of concern: Demographic change

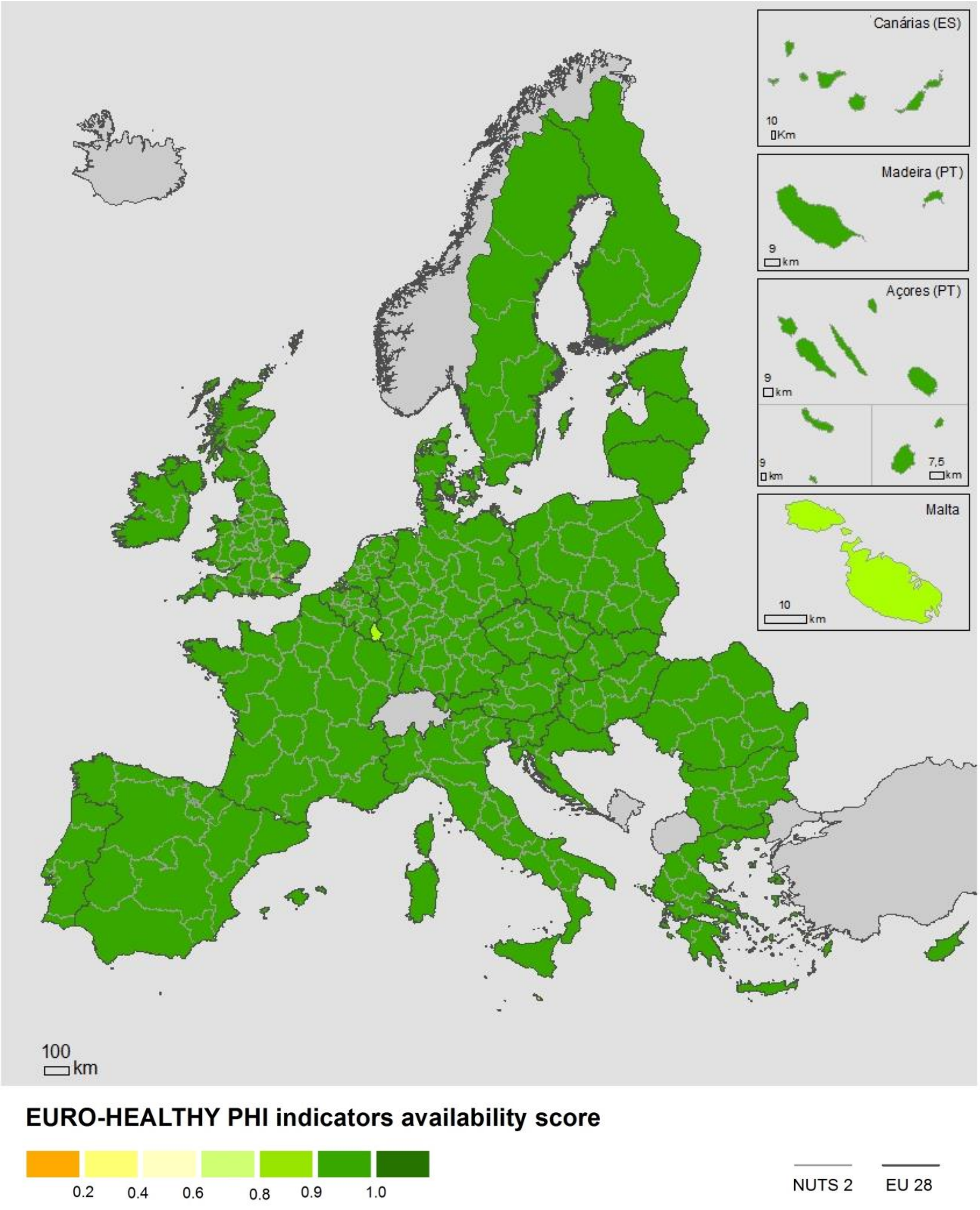

Regional availability score by area of concern: Lifestyle and health behaviours

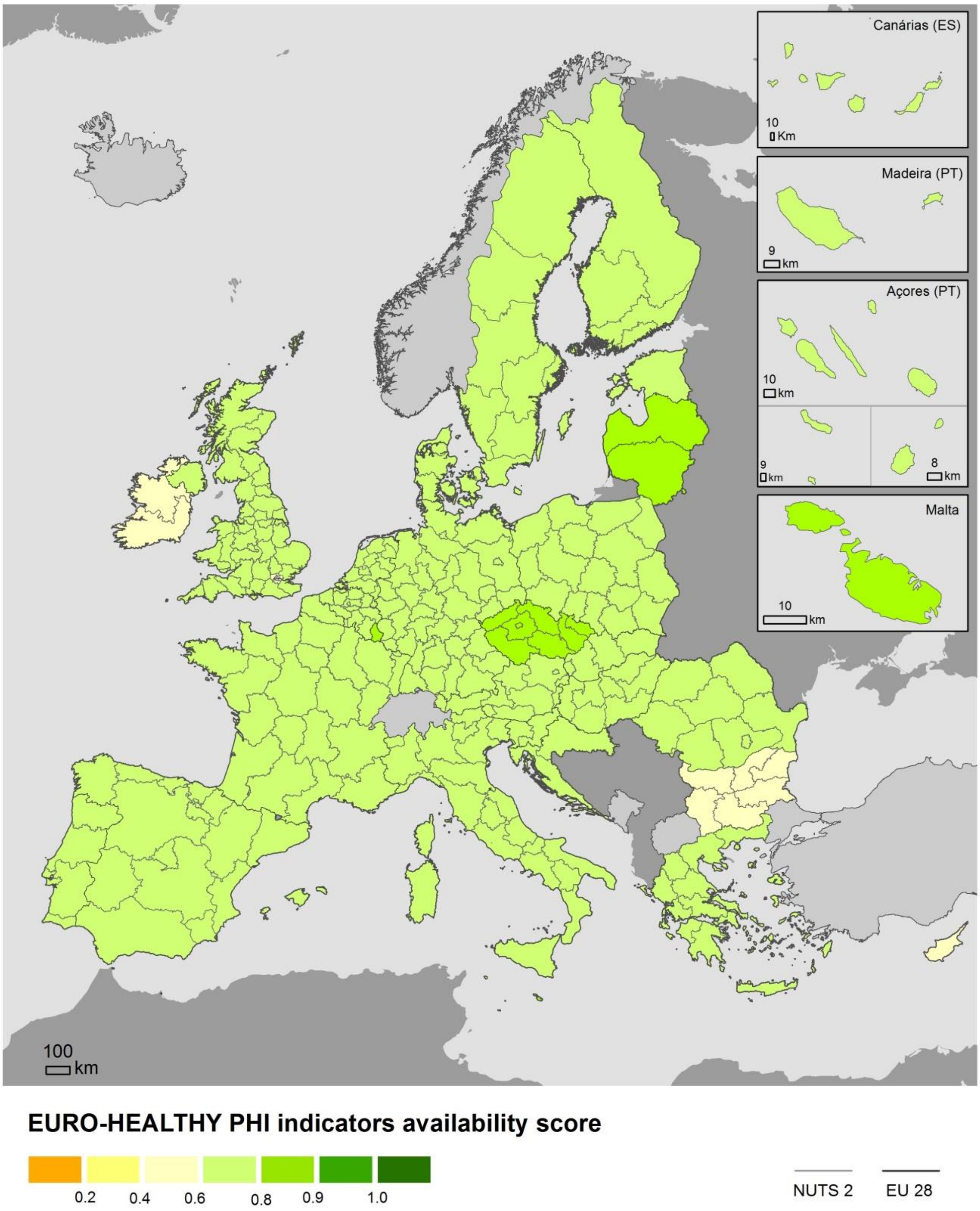

Regional availability score by area of concern: Physical Environment

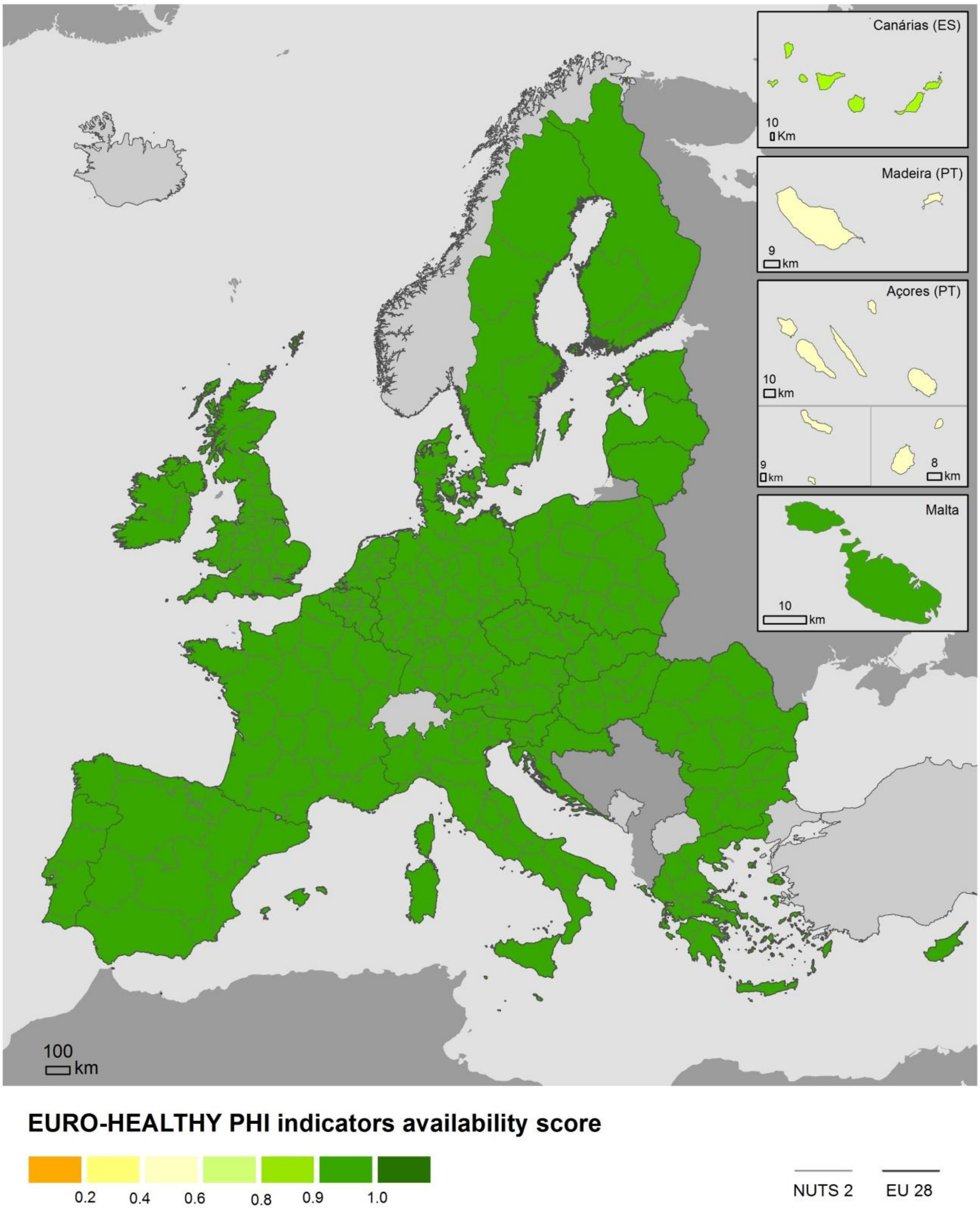

Regional availability score by area of concern: Built Environment

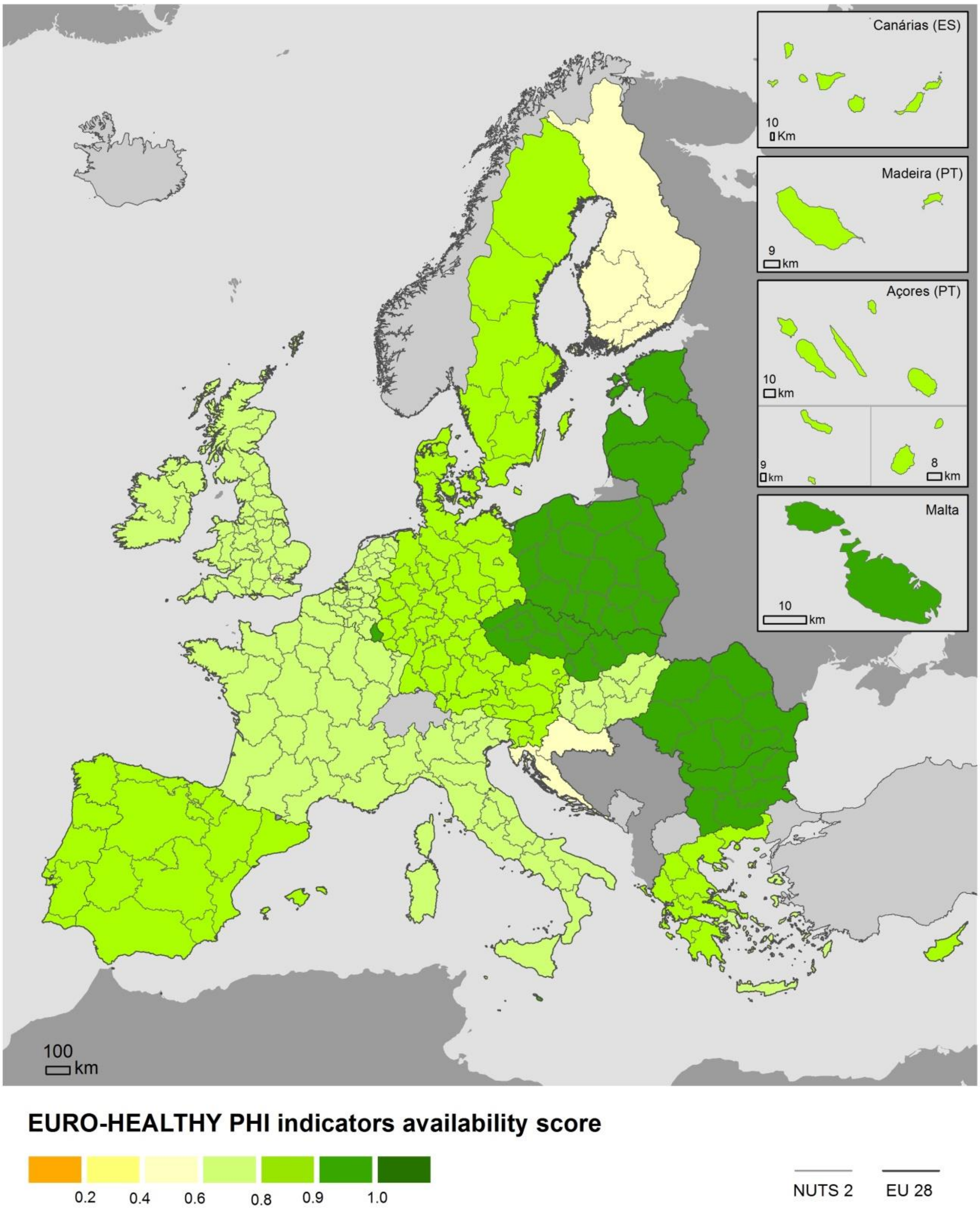

Regional availability score by area of concern: Road Safety

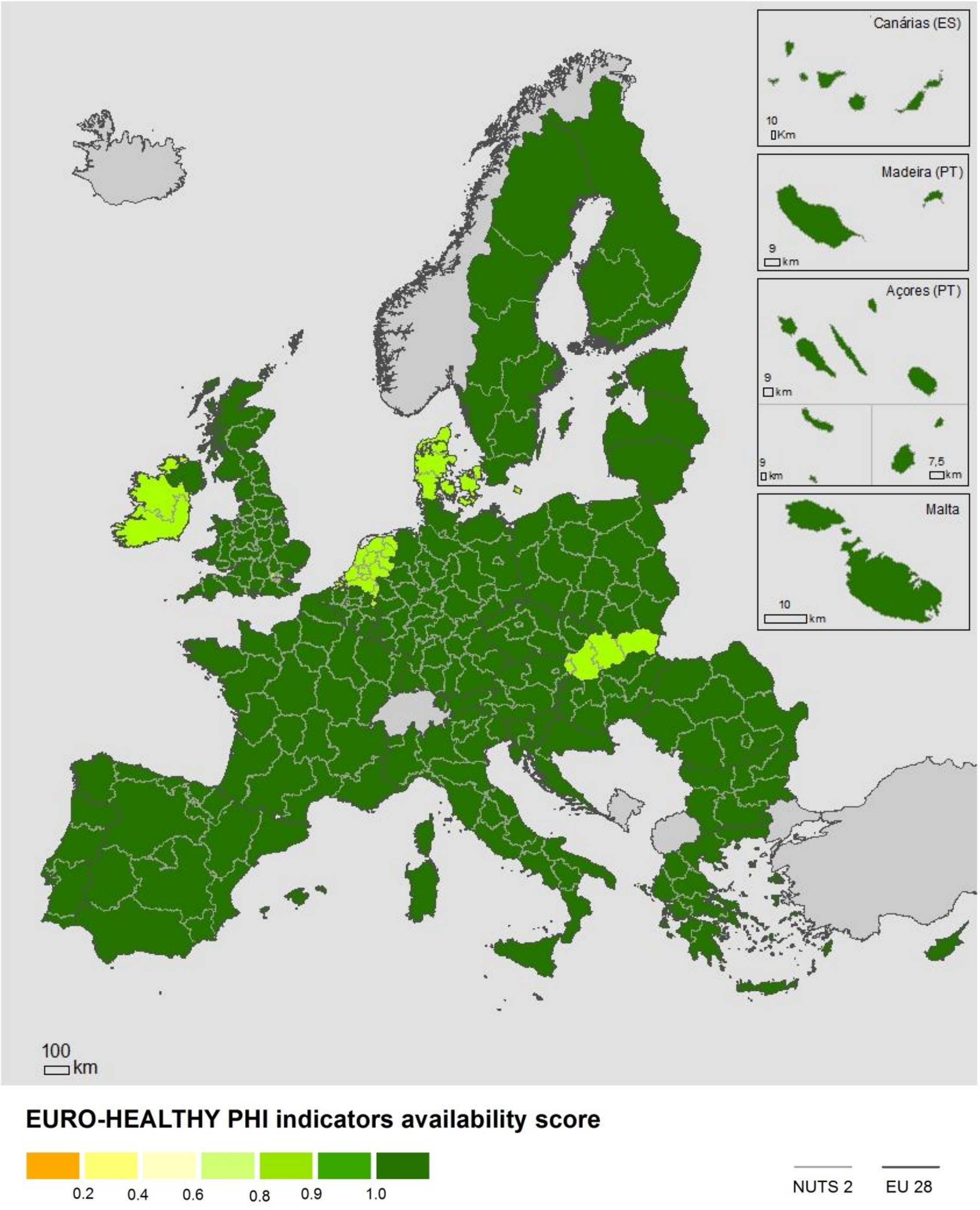

Regional availability score by area of concern: Healthcare resources and expenditure

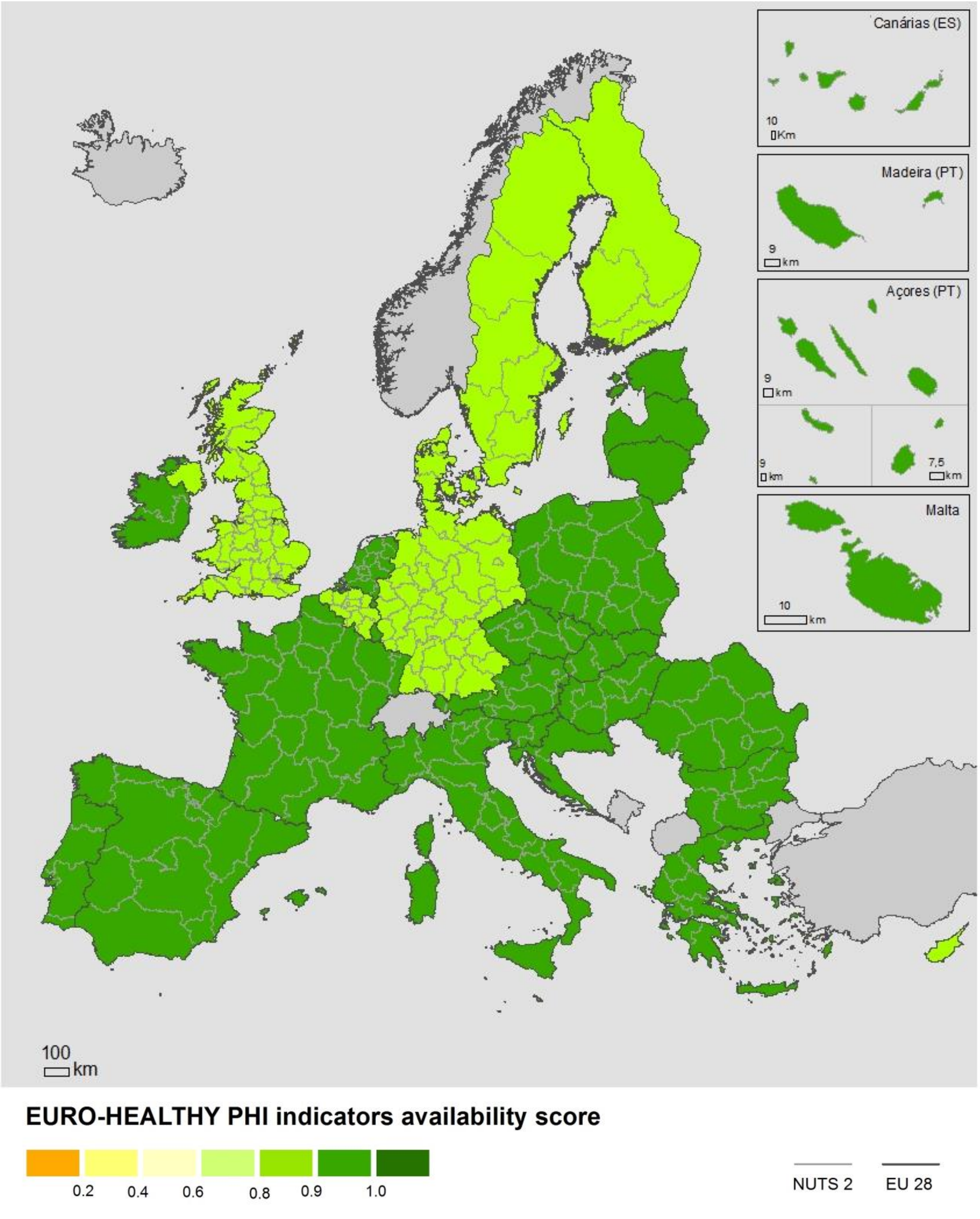

Regional availability score by area of concern: Healthcare performance

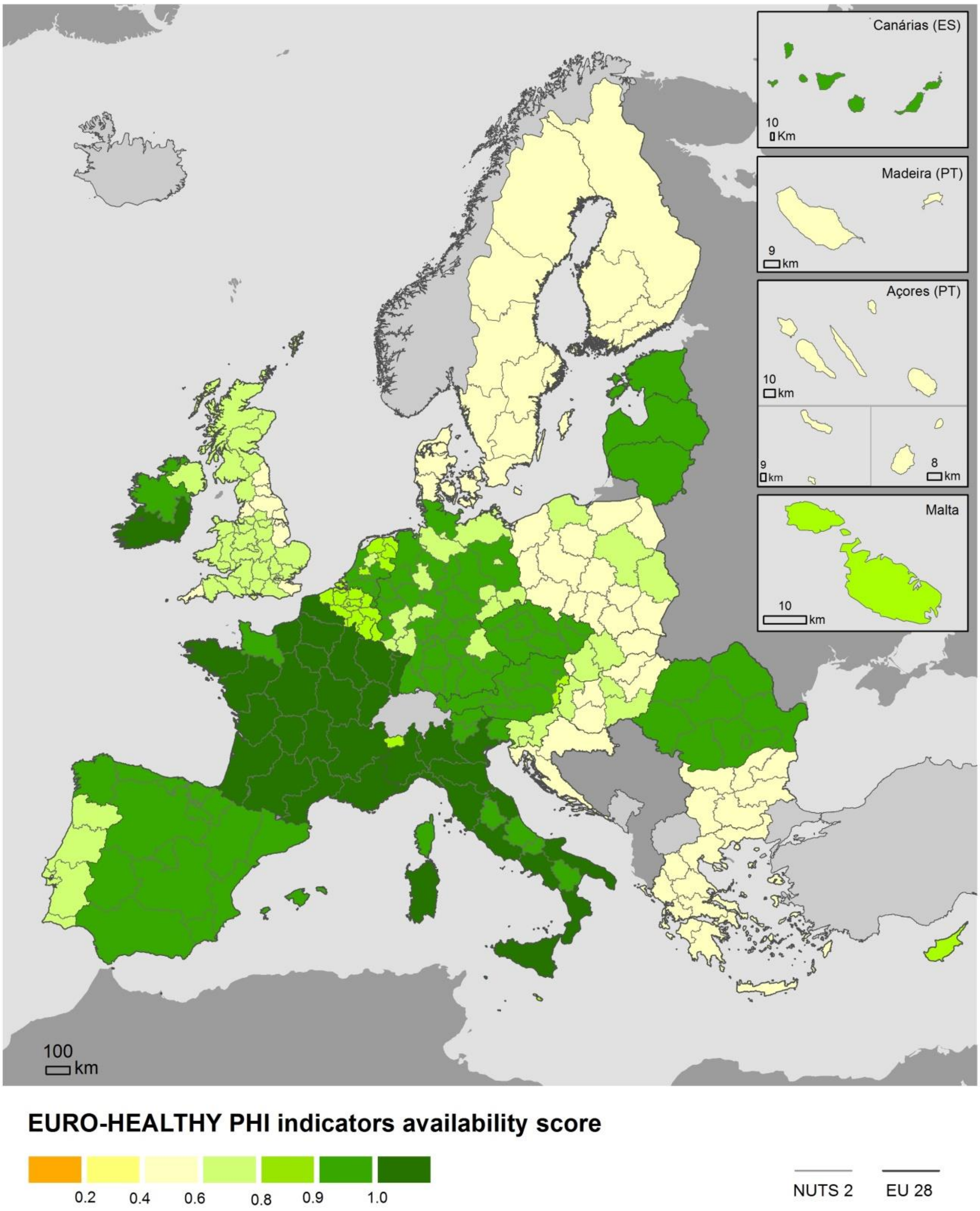

Regional availability score by area of concern: Health Outcomes

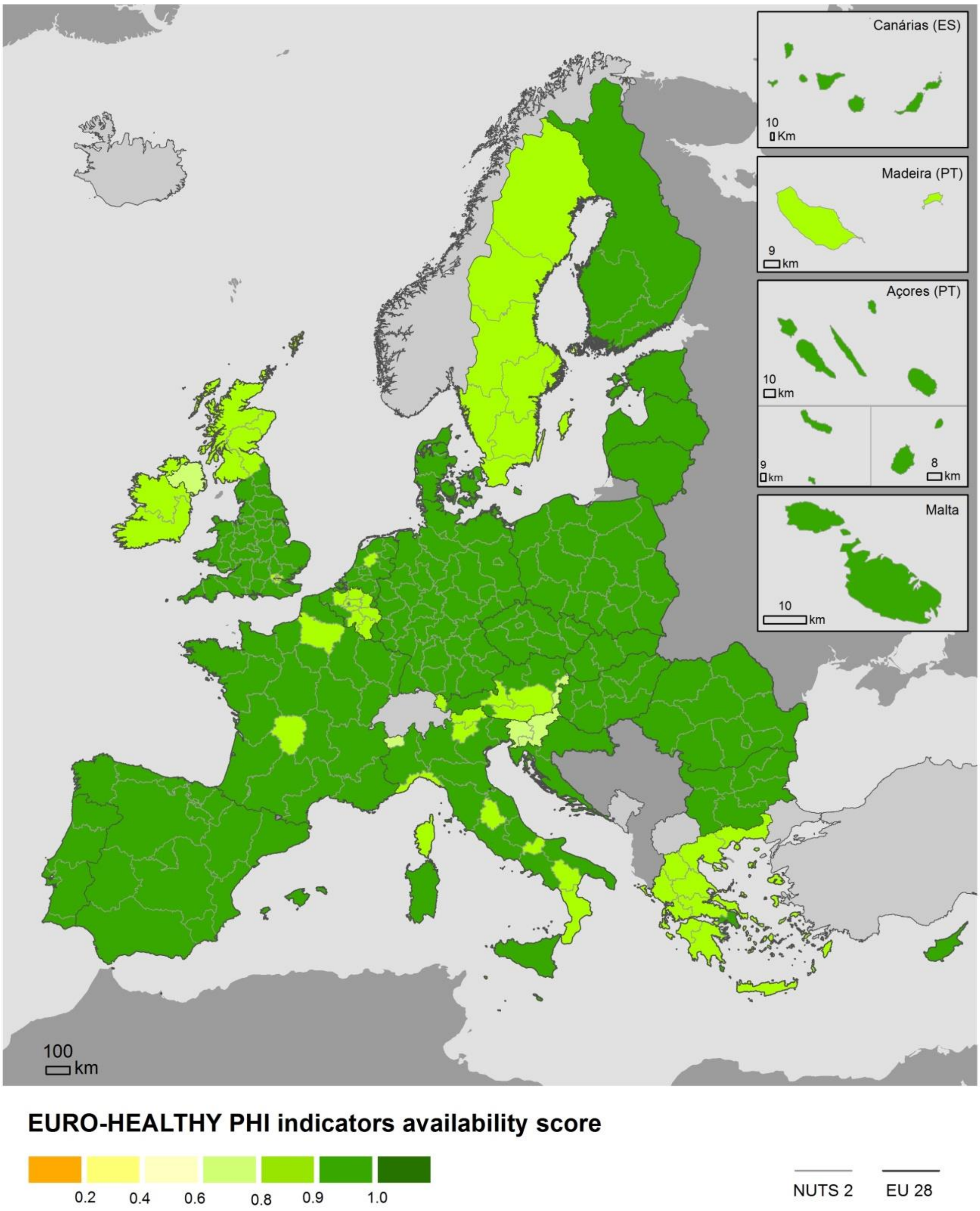

Supplement: Supplementary file 3 — Map of regional availability score in the EU, by area of concern. Figure with the regional availability score of each area of concern. (PDF 1870 kb) [file 12963_2019_188_MOESM3_ESM.pdf]
